# An Adaptive Feature Learning Model for Sequential Radar High Resolution Range Profile Recognition

**DOI:** 10.3390/s17071675

**Published:** 2017-07-20

**Authors:** Xuan Peng, Xunzhang Gao, Yifan Zhang, Xiang Li

**Affiliations:** College of Electronic Science and Engineering, National University of Defense Technology, Changsha 410073, China; gaoxunzhang@nudt.edu.cn (X.G.); zyifannudt@163.com (Y.Z.); lixiang01@vip.sina.com (X.L.)

**Keywords:** radar HRRP recognition, restricted Boltzmann machine, feature learning

## Abstract

This paper proposes a new feature learning method for the recognition of radar high resolution range profile (HRRP) sequences. HRRPs from a period of continuous changing aspect angles are jointly modeled and discriminated by a single model named the discriminative infinite restricted Boltzmann machine (Dis-iRBM). Compared with the commonly used hidden Markov model (HMM)-based recognition method for HRRP sequences, which requires efficient preprocessing of the HRRP signal, the proposed method is an end-to-end method of which the input is the raw HRRP sequence, and the output is the label of the target. The proposed model can efficiently capture the global pattern in a sequence, while the HMM can only model local dynamics, which suffers from information loss. Last but not least, the proposed model learns the features of HRRP sequences adaptively according to the complexity of a single HRRP and the length of a HRRP sequence. Experimental results on the Moving and Stationary Target Acquisition and Recognition (MSTAR) database indicate that the proposed method is efficient and robust under various conditions.

## 1. Introduction

In the radar automatic target recognition (RATR) community, recognition techniques based on radar high-resolution range profile (HRRP) have been widely studied [1,2,3,4,5]. An HRRP can be understood as a result of “scanning” the target from the direction of the radar line-of-sight, it contains discriminative features of the target such as the size and the distribution of scattering centers, etc. The common scheme for HRRP recognition is firstly to divide the full target-radar aspect angles into several stationary areas (also named “frames”) [2,5,6,7,8]. After that, target detection [9,10,11,12] is performed to select the region of interest in an HRRP. Finally, the HRRPs of each frame are further processed for recognition, which is also called feature extraction. Common feature extraction techniques include HRRP templates [13], HRRP stochastic modeling [2,3,5], time-frequency transform features [14,15], transform invariant features [16,17]. All these feature extraction techniques have their own advantages and disadvantages, none of them is optimal for target recognition.

Since the information of the target provided by a single HRRP is limited, utilizing sequential HRRP from multiple target-radar aspect angles can be a better choice for recognition. To make use of the spatial or temporal dependence in a sequence, HMM is often utilized for several sequential problems, such as sequential event detection in wireless sensor networks [18,19] and radar HRRP sequence recognition [20,21,22], where each HMM “state” consists of a set of generally contiguous target-sensor orientations over which the HRRP statistics are relatively stationary, and the statistical relationships in the HRRP sequence are described via the transition probabilities from one state to the next under HMM formulation. There are two paradigms of forming an HRRP sequence. One is done by continuously receiving HRRPs in a short period of time or aspect angles [21]. The angular sampling rate is dense, and the interval between adjacent HRRPs is often less than 0.1°. In this case, the HMM tries to model HRRP sequences in a single frame. Another way of forming an HRRP sequence is done by receiving HRRPs from different aspect-frames [20,22]. The sampling interval is often larger than 3° in this case, and the HMM tries to model the transitions between frames. The problem for the HMM is that it can only represent local dependencies between states, and it is not efficient to deal with high dimensional data, preprocessing of HRRP using the feature extraction techniques mentioned above is still required beforehand.

Modeling high dimensional sequential data has also been studied for decades in the machine learning community. Recently, the restricted Boltzmann machines (RBMs) have achieved great success in capturing spatial or temporal patterns in different types of data [23,24,25]. More specifically, an RBM is a bipartite graphical model that uses a layer of “hidden” binary variables or units to model the probability distribution of a layer of “visible” variables [26,27].The RBM and its various extensions have enjoyed much popularity for pattern analysis and generating due to the generality and flexibility of its graphical structure. However, for all types of RBMs, choosing a proper number of hidden units is essential but difficult. In order to deal with this issue, Côté [28] proposed a non-parametric model called the Infinite Restricted Boltzmann Machine (iRBM). The iRBM can automatically adjust the effective number of hidden units according to the training data, which is especially beneficial when the data size is changeable.

In this paper, we propose a new approach to sequential radar HRRPs recognition based on the iRBM. In order to make the iRBM to be capable of learning discriminative features, we modified the model to make it learning the joint probability distribution of sequential HRRPs and the corresponding target label. The HRRP data is converted from the SAR data of MSTAR [29]. HRRP sequences with different lengths are directly used to train the model. The maximal length covers about 1/3 of the full aspect. We didn’t train a full-aspect model for the reason that in real recognition scenarios, it is difficult to acquire HRRPs from all aspect angles, but it is much easier to acquire HRRPs from a fragment of aspect angles. The influence of HRRP sequences with different angular sampling rates at test phase has also been studied to investigate the robustness of our method. The features of the proposed method can be summarized as follows:

(a) It is an end-to-end model of which the input is the raw HRRP sequence and the output is the target class. Feature extraction and target classification are done in a single model.
(b) The model can efficiently capture the global pattern in a HRRP sequence, which is more powerful than other dynamic models such as the HMM.
(c) It is an adaptive model which can automatically decide the model complexity according to the complexity of HRRPs and the length of HRRP sequence.

The rest of the paper is summarized as follows: in Section 2, the RBM and iRBM are briefly introduced as a preparation for the proposal of the method. In Section 3, the proposed model for sequential HRRP recognition is presented in detail, followed by the training method for the model in Section 4. After that, several experiments on the MSTAR dataset have been performed to evaluate our model under various recognition scenarios in Section 5. Finally, we conclude our work in Section 6.

For convenience of reading, some notations for variables and equations in the paper are listed as follows:

(1) All one-dimensional variables are formatted in italic;
(2) All vectors and matrices are formatted in boldface;
(3) p(x) represents the probability distribution of x.

## 2. Preliminaries

### 2.1. Restricted Boltzmann Machines

**Figure 1:**
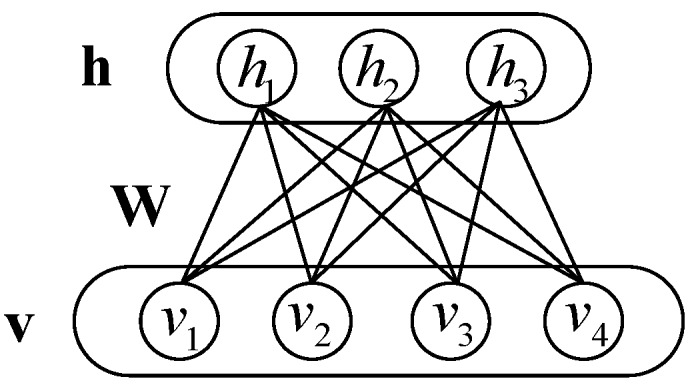
Graphical structure of the RBM.

The RBM is a bipartite graphic model, which means that it contains two layers, the visible layer and the hidden layer. The visible layer consists of the visible vector $v=\left[v_{1},v_{2},\cdots ,v_{D}\right]$ representing the observable data, the hidden layer consists of the hidden vector $h=\left[h_{1},h_{2},\cdots ,h_{N}\right]$. And each unit of the hidden layer is connected with all the units of visible layer and vice versa, while there is no connection within each layer. Both v and h are binary vectors. The corresponding graphical model for the RBM is illustrated in Figure 1.

The RBM represents a joint probability distribution p(v,h) with the so-called energy function E(v,h) defined below [26]:

(1) $$E\left(v,h\right)=-h^{T}Wv-b^{T}v-c^{T}h$$

where Θ={b,c,W} is the set of parameters. And p(v,h) is given as follows:

(2) $$p\left(v,h\right)=\frac{e^{-E\left(v,h\right)}}{Z}$$

where $Z=\sum_{v^{\prime }}\sum_{h^{\prime }}e^{-E\left(v^{\prime },h^{\prime }\right)}$ is the partition function which ensures that Equation (2) is a valid probability distribution.

An RBM can be used to model the distribution of the observed data p(v) by learning from the training data $v_{t}$. The training is done in an unsupervised manner. After the model is trained, we can sample new data from the model. The hidden layer can be also treated as a representation of the data using p(h|v). In this case, an RBM is treated as a feature extractor which preprocesses the data for other purposes such as classification [30].

### 2.2. Infinite Restricted Boltzmann Machines

The iRBM [28] is proposed to settle the difficulty of choosing proper number of hidden units for the RBMs, it can effectively adapt its capacity as training progresses. The iRBM is an extension of the ordinary RBM, which mixes infinite number of RBMs with different number of hidden units from 1 to ∞, and all the RBMs choose the hidden units in sequence from the same set. The energy function of the iRBM is defined as follows:

(3) $$E\left(v,h,z\right)=-v^{T}b^{v}-\sum_{i=1}^{z}h_{i}\left(W_{i\cdot }v+b_{i}^{h}\right)-\beta_{i}$$

where, v is the D dimensional visible vector representing the observable data. $h_{i}$ is the $i^{\mathrm{th}}$ element of the infinite-dimensional hidden vector h. $\beta_{i}$ is the penalty for each selected hidden unit $h_{i}$. $W_{i\cdot }$ is the $i^{\mathrm{th}}$ row of weight matrix W connecting the visible units and the hidden units. $b^{v}$ is the visible units biases. $b_{i}^{h}$ is the $i^{\mathrm{th}}$ hidden unit bias. The random variable z∈N can be understood as the total number of hidden units being selected to participate in the energy function.

It should be noticed that for a given z, the value of the energy function is irrelevant for the dimensions of h from z+1 to ∞, which means that $h_{i}$ where i>z will never be activated. Thus, (3) has the same form with the energy function of ordinary RBM with z hidden units except the penalty $\beta_{i}$ which the latter does not have.

The joint probability over v, h and z is:

(4) $$p\left(v,h,z\right)=\frac{1}{Z}e^{-E\left(v,h,z\right)}$$

where:

(5) $$Z=\sum_{z^{\prime }=1}^{\infty }\sum_{v^{\prime }}\sum_{h^{\prime }\in H_{z^{\prime }}}e^{-E\left(v^{\prime },h^{\prime },z^{\prime }\right)}$$

and $H_{z}=\left\{h\in H|h_{k}=0\forall k>z\right\}$, where H is the set of all possible values h takes. Thus $H_{z}$ defines the legal values of h given z.

The hidden units are selected in sequence as z takes the value from 1 to ∞, and if the penalty $\beta_{i}$ is chosen properly, an infinite pool of hidden units can be achieved. A way to parameterize $\beta_{i}$ is suggested in [28], which is $\beta_{i}=\beta \ln\left(1+e^{b_{i}^{h}}\right)$. This will ensure the partition function Z is convergent as long as β>1 and the number of hidden units having non-zero weights and biases is always finite. In this paper, we just used the same value $\beta_{i}=1.01\times \ln2$ in all the experiments, as we found that the performance of the model is robust to the choices of $\beta_{i}$. The major effect of $\beta_{i}$ is on learning speed rather than the final performance.

It needs to be pointed out here that, for both RBMs and iRBMs, exactly computing p(v) is intractable as computing the partition function *Z* involves summing all possible states of hidden units. However, inferring h from v or conversely is easy. Thus, the model can be used to draw new samples $v^{1}$, $v^{2}$, ⋯, $v^{T}$ efficiently by conducting Gibbs sampling ($h^{0}\to v^{1}\to h^{1}\to v^{2}\cdots h^{T-1}\to v^{T}$).

## 3. The Proposed Model

The iRBM has a nice property of adaptively learning the numbers of hidden units, which is especially beneficial when the dataset size is changeable. However, it can only perform unsupervised learning, there is no guarantee that the learnt features are helpful for discrimination. In this section, a modified version of iRBM is proposed which can jointly learn the distribution of the HRRP sequence $\left(v_{1},\cdots ,v_{L}\right)$ with sequence length *L*, and its corresponding label y.

By introducing the label of data into the energy function (3), a new model representing the joint probability of $\left(v_{1},\cdots ,v_{L}\right)$, h, y and z is achieved, which is:

(6) $$p\left(v_{1},\cdots ,v_{L},h,y,z\right)=\frac{1}{Z}e^{-E\left(v_{1},\cdots ,v_{L},h,y,z\right)}$$

where the modified energy function is:

(7) $$E\left(v_{1},\cdots ,v_{L},h,y,z\right)=-\sum_{l=1}^{L}v_{l}^{T}b_{l}-e_{y}{}^{T}d-\sum_{i=1}^{z}h_{i}\left(\sum_{l=1}^{L}W_{i\cdot }^{l}v_{l}+U_{i\cdot }e_{y}+c_{i}\right)-\beta_{i}$$

where, $v_{l}$ is the $l^{\mathrm{th}}$ HRRP vector of the sequence. $e_{y}={\left(1_{i=y}\right)}_{i=1}^{C}$ is the “one hot” representation of the label y∈{1,2,⋯,C}, and C is the total number of classes. d is the label bias vector with the same dimension to $e_{y}$. $W_{i\cdot }^{l}$ is the $i^{\mathrm{th}}$ row of weight matrix $W^{l}$ connecting the $l^{\mathrm{th}}$ HRRP vector and the hidden units h. $U_{i\cdot }$ is the $i^{\mathrm{th}}$ row of the weight matrix U which connects h and $e_{y}$.

By marginalizing out h and z, we get the marginal distribution $p\left(v_{1},\cdots ,v_{L},y\right)$ as follows:

(8) $$p\left(v_{1},\cdots ,v_{L},y\right)=\frac{1}{Z}\sum_{z=1}^{\infty }\sum_{h\in H_{z}}e^{-E\left(v_{1},\cdots ,v_{L},h,y,z\right)}=\frac{1}{Z}\sum_{z=1}^{\infty }e^{-F\left(v_{1},\cdots ,v_{L},y,z\right)}$$

where:

(9) $$F\left(v_{1},\cdots ,v_{L},y,z\right)=-\sum_{l=1}^{L}v_{l}^{T}b_{l}-e_{y}{}^{T}d-\sum_{i=1}^{z}\ln\left(1+\exp\left(\sum_{l=1}^{L}W_{i\cdot }^{l}v_{l}+U_{i\cdot }e_{y}+c_{i}\right)\right)-\beta_{i}$$

**Figure 2:**
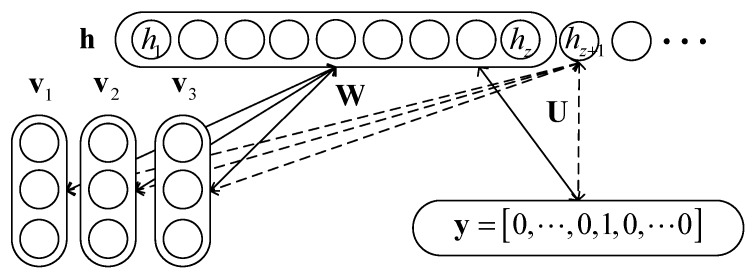
Graphical structure of the Dis-iRBM for sequential HRRP.

We name this model the discriminative infinite restricted Boltzmann machine (Dis-iRBM). The graphical structure of the proposed model is demonstrated in Figure 2.

The Dis-iRBM has a self-contained structure for supervised learning just as the ClassRBM [31] does. Furthermore, it learns features more flexibly. It can be trained discriminatively or generatively [31]. In the following section, a hybrid training objective combining discriminative and generative training objective together is proposed to learn the parameters of the model.

## 4. Learning the Parameters of the Model

The training objective to learn the parameters is a hybrid training objective combining the generative and the discriminative training objective similar to [31], and is given below:

(10) $$\begin{array}{c} f_{\mathrm{hybrid}}\left(\Theta ,D_{\mathrm{train}}\right)=-\frac{1}{\left|D_{\mathrm{train}}\right|}\left(\left(1+\alpha \right)\sum_{n=1}^{\left|D_{\mathrm{train}}\right|}\ln p\left(y_{n}|v^{n}\right)+\alpha \sum_{n=1}^{\left|D_{\mathrm{train}}\right|}\ln p\left(v^{n}\right)\right)\\ =\frac{1}{\left|D_{\mathrm{train}}\right|}\left(\left(1+\alpha \right)f_{\mathrm{dis}}\left(\Theta ,D_{\mathrm{train}}\right)+\alpha f_{\mathrm{gen}}\left(\Theta ,D_{\mathrm{train}}\right)\right) \end{array}$$

where, $D_{\mathrm{train}}=\left\{\left(v^{n},y_{n}\right)\right\}$ is the set of training examples, and $v^{n}=\left(v_{1}^{n},\cdots ,v_{L}^{n}\right)$. The way to construct training examples of HRRP sequences will be explicitly introduced in Section 5. $f_{\mathrm{dis}}\left(\Theta ,D_{\mathrm{train}}\right)=-\sum_{n=1}^{\left|D_{\mathrm{train}}\right|}\ln p\left(y_{n}|v^{n}\right)$ corresponds to the discriminative part modeling $p\left(y_{n}|v^{n}\right)$, and $f_{\mathrm{gen}}\left(\Theta ,D_{\mathrm{train}}\right)=-\sum_{n=1}^{\left|D_{\mathrm{train}}\right|}\ln p\left(v^{n}\right)$ corresponds to the generative part modeling the inputs $p\left(v^{n}\right)$ only. α>0 controls the proportion of each part. The second part can be thought of as a model regularization term.

The learning of the generative part $f_{\mathrm{gen}}\left(\Theta_{t},D_{\mathrm{train}}\right)$ is identical with the learning of the iRBM introduced in Section 2. The Contrastive Divergence (CD) and the Persistent Contrastive Divergence (PCD) [32,33] can be directly used to compute the gradients. The approximated gradient for the generative part is given below:

(11) $$\frac{\partial }{\partial \theta }f_{\mathrm{gen}}\left(\Theta_{t},D_{\mathrm{train}}\right)\approx \frac{1}{\left|D_{\mathrm{train}}\right|}\sum_{n=1}^{\left|D_{\mathrm{train}}\right|}\left(\frac{\partial }{\partial \theta }F\left(v^{n},z^{\mathrm{pos}}\right)-\frac{\partial }{\partial \theta }F\left(v^{\mathrm{neg}},z^{\mathrm{neg}}\right)\right)$$

where, $z^{\mathrm{pos}}$ is sampled from $p\left(z|v^{n}\right)$, and $v^{\mathrm{neg}},z^{\mathrm{neg}}$ are sampled from $p\left(v^{n},z\right)$ by K-step Gibbs sampling, the detail of learning a iRBM is provided in [24].

In order to calculate the gradient of the discriminative part $f_{\mathrm{dis}}\left(\Theta_{t},D_{\mathrm{train}}\right)$, the conditional probability $p\left(y|v^{n}\right)$ is derived as follows:

(12) $$p\left(y|v^{n}\right)=\frac{p\left(y,v^{n}\right)}{p\left(v^{n}\right)}=\frac{\sum_{z=1}^{\infty }e^{-F\left(v^{n},y,z\right)}}{\sum_{z=1}^{\infty }\sum_{y^{\prime }}e^{-F\left(v^{n},y^{\prime },z\right)}}=\frac{\sum_{z=1}^{\infty }e^{-G\left(y,z|v^{n}\right)}}{\sum_{z=1}^{\infty }\sum_{y^{\prime }}e^{-G\left(y^{\prime },z|v^{n}\right)}}$$

where:

(13) $$G\left(y,z|v^{n}\right)=-d_{y}-\sum_{i=1}^{z}\ln\left(1+\exp\left(\sum_{l=1}^{T}W_{i\cdot }^{l}v_{l}^{n}+U_{i\cdot }e_{y}+c_{i}\right)\right)-\beta_{i}$$

By taking Equations (12) and (13) into the discriminative part of (11), the gradient of $f_{\mathrm{dis}}\left(\Theta_{t},D_{\mathrm{train}},t\right)$ is derived as follows:

(14) $$\frac{\partial }{\partial \theta }f_{\mathrm{dis}}\left(\Theta ,D_{\mathrm{train}}\right)=\frac{1}{\left|D_{\mathrm{train}}\right|}\sum_{n=1}^{\left|D_{\mathrm{train}}\right|}\frac{\partial F\left(y_{n}|v^{n}\right)}{\partial \theta }-\sum_{y^{\prime }}p\left(y^{\prime }|v^{n}\right)\frac{\partial F\left(y^{\prime }|v^{n}\right)}{\partial \theta }$$

where:

(15) $$F\left(y|v^{n}\right)=-\ln\sum_{z=1}^{\infty }\left(e^{-G\left(y,z|v^{n}\right)}\right)$$

and

(16) $$p\left(y^{\prime }|v^{n}\right)=\frac{e^{-F\left(y|v\right)}}{\sum_{y^{\prime }}e^{-F\left(y^{\prime }|v^{n}\right)}}$$

The gradients $\partial F\left(y_{n}|v^{n}\right)/\partial \theta$ can be exactly computed, as shown in the Appendix. However, this involves computing gradients for infinite many parameters. To avoid this issue, we only compute the gradients for parameters of first $M_{t}$ hidden units whose parameters are non-zero at gradient descent step t, and leave all the remaining parameters to be 0. The complexity of computing (16) is $O\left(M_{t}^{2}D+M_{t}^{2}C\right)$.

The maximum number of activated hidden units $M_{t}$ changes gradually during training. Practically, if the Gibbs sampling chain ever samples a value of z larger than $M_{t}$, we clamp it to $M_{t}+1$. This avoids filling large memory for a large (but rare) value of z. The training procedure for the proposed recognition model is summarized as follows:

Step 1: Divide the training dataset of HRRPs into several aspect frames;
Step 2: Construct the sequential HRRP training data with length *L* by *L* HRRPs from *L* adjacent aspect frames, and each frame provides one HRRP. See the details in Section 5.
Step 3: Train several different models on the data with different lengths *L* using the training method described above.

After the model is trained, a new sample of the sequential HRRP data $v^{\prime }=\left(v_{1}^{\prime },\cdots ,v_{L}^{\prime }\right)$ is obtained using the same aspect frame detecting technique in the training phase. And the likelihood of each label conditioned on the new data is computed using the trained model with the same sequence length *L*. Finally, it is assigned to the class y* according to the following decision rule:

(17) $$y*=\arg\;\underset{y\in \left\{1,\cdots ,C\right\}}{\max}\;p\left(y|v^{\prime }\right)$$

As mentioned above, the complexity of computing (16) or (17) is $O\left(M_{t}^{2}D+M_{t}^{2}C\right)$. $D=L\times D_{0}$, and $D_{0}$ is the number of dimensions of a single HRRP, of which the order of magnitude is 100. The sequence length *L* is usually less than 50. And the class numbers *C* has a magnitude order of about 10. Thus the approximate computational complexity for a typical recognition problem is $O\left(5000\times M_{t}^{2}\right)$, which is a quadratic function of the model size $M_{t}$. The computation can be performed in real-time as long as $M_{t}$ is not extremely large.

## 5. Experimental Results

In this section, several experiments on the MSTAR dataset have been performed to evaluate the proposed recognition model. Firstly, the way of arranging the training and testing HRRP sequences have been introduced. Then, two kinds of experiments have been done on this dataset. The first experiment investigated the influence of HRRP sequence length on recognition performance. The second experiment investigated the robustness of the proposed model when the angular sampling rates at test phase were different from that at training phase. To accelerate the learning of Dis-iRBMs, we used a new training strategy referred as “RP” training [34] to train the Dis-iRBMs in all experiments.

### 5.1. The Dataset

SAR images of three types of targets (the T-72 main battle tank, the BMP-2 armored personnel carrier, and the BTR-70 armored personnel carrier) in MSTAR were used to construct the sequential HRRP dataset. The SAR images of the training set were taken at a depression angle of 17°, while the testing set depression angle is 15°. SAR images of all targets covered the full aspect angles, and the training and the testing images of the same vehicle at the same aspect angle are different. Variants (different serial number) of the three targets were also used in the testing set to evaluate the generalization ability of the recognition method. The dataset of three-target problem is briefly illustrated in Table 1. The size of the training and testing sets of SAR images is 698 and 1365, respectively.

We converted each SAR image into 10 mean HRRPs, thus the training set and testing set contain 6980 HRRPs and 13,650 HRRPs respectively. In many literatures, the clutter is removed to get “clean” HRRPs, while we directly used the raw HRRPs, the only preprocessing were normalizing the magnitude of each HRRP to its total energy. This setting could make the experiments more closed to real recognition scenarios. We divided the 360° of aspect angles into 50 aspect frames uniformly, each frame covers 7.2°, this division of aspect frames may not be optimized, but is similar to that described in [20], which allow us to conveniently compare between these two methods. Suppose the total number of HRRPs for a target is *N*, then each aspect frame contains about *N*/50 HRRPs. The following steps were taken to construct the sequential HRRP data:

Step 1: The first HRRPs from aspect frame 1 to aspect frame L are chosen to form an HRRP sequence with length L. Slide one unit to the right to choose the second HRRPs from the same frames to form another HRRP sequence. Repeat this procedure until the end of each frame;
Step 2: Slide one frame to the right and repeat step 1;
Step 3: Repeat step 2 until the end of all aspect frames. If the sequence starts after (50−L) th frame, then the first L−1 frames are cyclically used one by one to form complete sequences.

**Figure 3:**
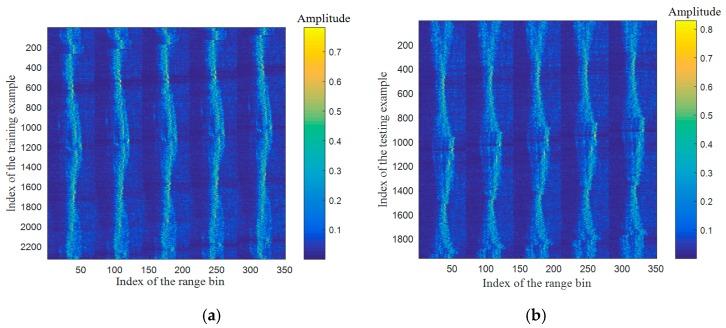
Some examples of training and testing HRRP sequences, (**a**) training; (**b**) testing.

The total number of HRRP sequences is exactly *N* by constructing the data in this way, and each HRRP sample can appear at anywhere of the sequence. Part of training set and testing set are illustrated in Figure 3, where the sequence length L=5.

### 5.2. Experiment 1: Investigating the Influence of HRRP Sequence Length on Recognition Performance

**Figure 4:**
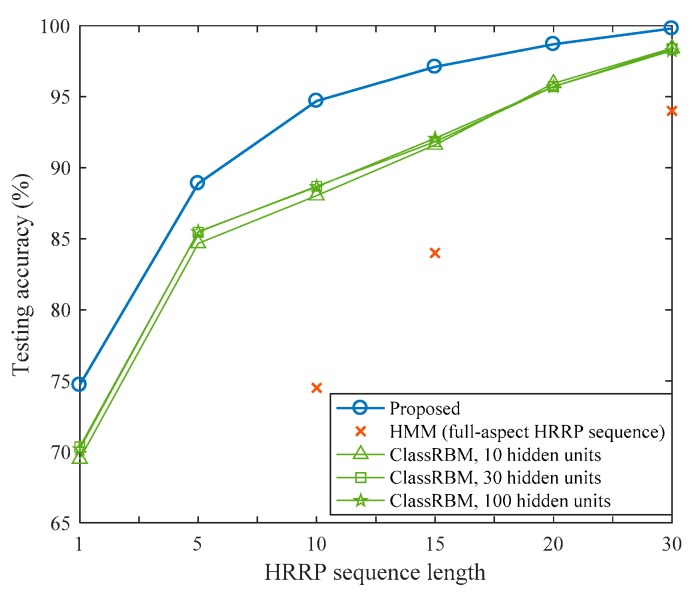
Recognition performance on models trained with different sequence length data. The result of HMM is provided by [20] in which a full-aspect HMM containing 55 states (aspect frames) was trained.

In this sub-section, five individual models were trained by the data with different sequence lengths (*L* = 1, 5, 10, 15, 20, 30). We also trained ClassRBMs with different hidden layer sizes as comparisons to the proposed method. At the testing phase, the data has the same angular sampling rate and sequence length with the training data. In this case, each model is supposed to have the best performance, thus comparison between different models is fair. The recognition performance of each model is shown in Figure 4.

As shown in Figure 4, the longer the sequence is, the higher the test accuracy can achieve. This is not surprising as multiple HRRP contains more information about the target than a single HRRP. The proposed model can efficiently utilize more complicated information owing to the strong representation power and adaptive feature learning property. Reference [20] used a similar way of constructing the sequence data which resulted in 55 aspect frames. They trained HMMs on the full-aspect HRRP sequences (*L* = 55), and the maximal sequence length for test was 30 with the same angular sampling rate in the test phase. By this setting, they reached a result of 94%. But the sequence length is too long (covering more than half of the aspect angles), which often cannot be fulfilled in real recognition scenarios. Our model outperforms [20] when the length is longer than 10. The best performance in this experiment is 99.8% when the sequence length *L* = 30. This result is ideal as the test and training angular sampling rates are identical and the sequence length is long enough. As for ClassRBM, the number of hidden units cannot be learnt, it has to be specified before training. We tried three different hidden layer sizes ($N_{h}$ = 10, 30, 100). The performances of ClassRBMs indicate that increasing the model complexity would not necessarily result in increasing the recognition performance. And a too large model is also more likely to over fit the data. Thus it is essential to decide a proper size of the model for better generalization. The confusion matrix achieved by the model train on HRRP sequence with *L* = 15 is illustrated in Table 2.

**Figure 5:**
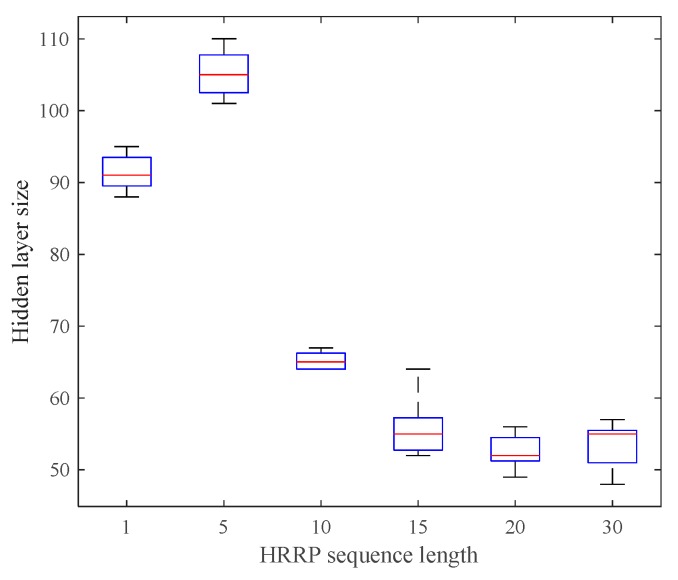
Effective hidden layer sizes of Dis-iRBMs trained on the data with different sequence lengths.

To validate the adaptive property of the proposed model, we repeated the training procedure on each sequence length five times. The average model size over five trials on each sequence length is illustrated in Figure 5. We can see from the figure that the number of hidden units went down and became stable at 50~60 when the sequence length was larger than 15. This is not surprising. As the sequence becomes longer, the visible data become more structured and regular. The hidden units can discover global patterns more efficiently and share it with each other, which results in the need of less number of hidden units.

**Figure 6:**
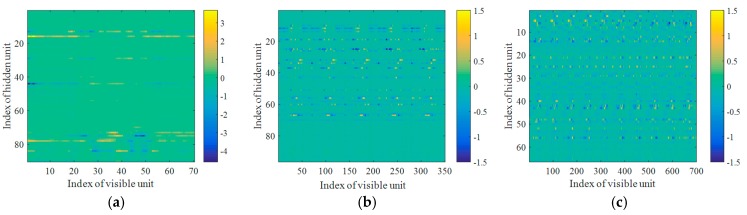
Weight matrices W or filters learnt by Dis-iRBMs, (**a**) *L* = 1; (**b**) *L* = 5; (**c**) *L* = 10.

Figure 6 illustrates weight matrices W or filters learnt by Dis-iRBMs, where each row in the weight matrix represents a filter learnt by the corresponding hidden unit.

### 5.3. Experiment 2: Investigating Robustness of the Proposed Model on Alternative Angular Sampling Rates in Test Phase

**Figure 7:**
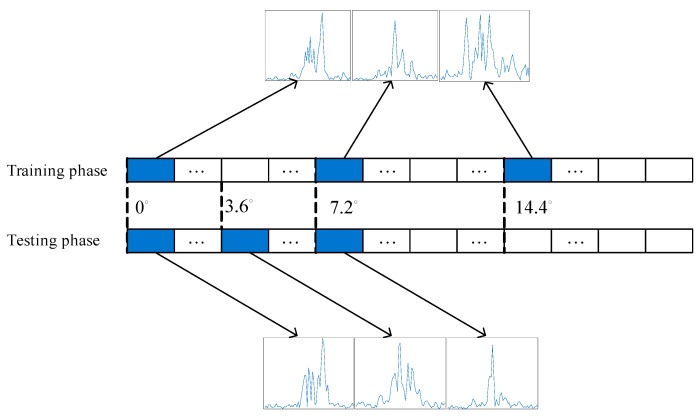
A illustration of the difference of angular sampling intervals between training and testing phase. Where the ratio between testing and training sampling intervals is 1/2.

In real recognition scenarios, it is difficult to ensure that the angular sampling rate in the test phase is identical to that in the training phase, especially when the relative pose of the target is unavailable. Thus it is essential for the recognition method to have some robustness to the change of the sampling rate. The difference in training and test angular sampling intervals is illustrated in Figure 7.

**Figure 8:**
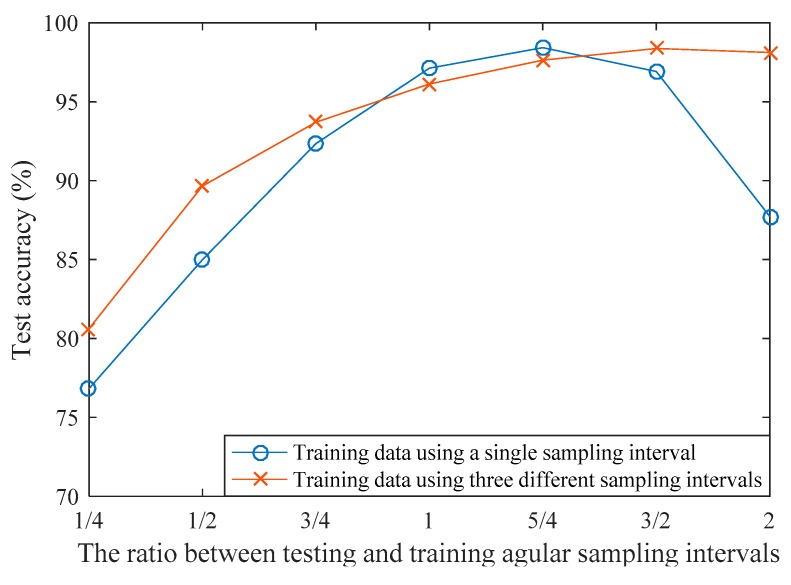
Recognition performance on different ratios between testing and training angular sampling intervals.

Here, we used the best model trained on the data with *L* = 15 and sampling interval *T*_0_ = 7.2° to evaluate this property. Several different testing datasets were constructed using different ratios between testing and training sampling intervals (1/4, 1/2, 3/4, 1, 5/4, 3/2, 2). The results are shown in Figure 8. The test accuracy ranges from 76.8% to 98.4%. In overall, the recognition performance is improved when the testing and training angular sampling intervals get closer to each other. Intuitively, the model will perform the best when sampling ratio is 1. However this is not the case in this experiment, the model performs the best when the ratio is 5/4. The reason of this unexpected result may come from the fact that, the whole aspect angles was uniformly divided into 50 frames. This division of frames may not be optimized, e.g., the evolution of statistic property of HRRP with respect to the aspect angle is often not uniform. Better performance will be achieved if more elaborate way of frame division is utilized. We also used an expanded training set containing three different angular sampling intervals (*T*_0_ = 7.2°, *T*_1_ = 3.6°, *T*_2_ = 14.4°) to train the model, and *T*_0_ = 7.2° was used as benchmark for comparing to the testing sampling intervals. Its test performance ranges from 80.6% to 98.4%. An interesting fact from this experiment is that, higher sampling interval is preferred for better recognition. For a single radar, it is not easy to sampling the HRRPs with large angular intervals. However, this condition can be fulfilled more easily if there exist multiple radars observing the target from different aspect angles.

## 6. Conclusions

This paper provides an approach for efficiently recognizing radar HRRP sequences. The proposed model has appealing properties of adaptive feature learning and capturing global features in a HRRP sequence. It is an end-to-end method which directly processes the raw HRRP sequence data and outputs the target label. It achieves high recognition accuracy when the sequence length covers more than 1/5 of the aspect angles, which outperforms the HMM using the HRRP sequences covering the whole aspect angles. It is more flexible and generalizes better than the ClassRBM. The model also shows some robustness to the change of angular sampling rate, the results with respect to angular sampling intervals also indicates that higher sampling interval might be preferred for better recognition. In the future, the proposed model can be improved in three directions. The first is trying to import area knowledge of the HRRP signal into the model to make it representing the HRRP more accurately. The second is studying the effects of angular sampling interval or frame division on model’s performance. The third is developing a model which can efficiently take different scales of relative angular speeds into account thus may enhance its robustness to the change of angular sampling rates in real recognition scenarios.

## Figures and Tables

**Table 1:** Training and testing set of SAR images for the three-target problem.

Training Set	Size	Testing Set	Size
T72 (Sn_132)	232	T72 (Sn_132)	196
		T72 (Sn_812)	195
		T72 (Sn_S7)	191
BTR70 (Sn_C71)	233	BTR70 (Sn_C71)	196
BMP2 (Sn_C9563)	233	BMP2 (Sn_C9563)	195
		BMP2 (Sn_C9566)	196
		BMP2 (Sn_C21)	196

**Table 2:** Confusion matrix of the best model (*L* = 15) for three targets problem.

True Inferred	BMP2	BTR70	T72	Accuracy (%)
BMP2	5613	45	212	95.62
BTR70	54	1906	0	97.24
T72	81	0	5731	98.61
Total accuracy(%)	97.13			

## Appendix A

Derivation of gradients (14) w.r.t. θ is presented as follows. Recall the Equation (14):

$$\frac{\partial }{\partial \theta }f_{\mathrm{dis}}\left(\Theta ,D_{\mathrm{train}}\right)=\frac{1}{\left|D_{\mathrm{train}}\right|}\sum_{n=1}^{\left|D_{\mathrm{train}}\right|}\frac{\partial F\left(y_{n}|v^{n}\right)}{\partial \theta }-\sum_{y^{\prime }}p\left(y^{\prime }|v^{n}\right)\frac{\partial F\left(y^{\prime }|v^{n}\right)}{\partial \theta }$$

where:

(A1) $$\begin{array}{cc} \frac{\partial F\left(y|v^{n}\right)}{\partial \theta } & =\frac{\partial }{\partial \theta }-\ln\sum_{z=1}^{\infty }\left(e^{-G\left(y,z|v^{n}\right)}\right)\\ & =-\frac{1}{\sum_{z=1}^{\infty }\left(e^{-G\left(y,z|v^{n}\right)}\right)}\frac{\partial }{\partial \theta }\sum_{z=1}^{\infty }\left(e^{-G\left(y,z|v^{n}\right)}\right)\\ & =\sum_{z=1}^{\infty }\frac{e^{-G\left(y,z|v^{n}\right)}}{\sum_{z=1}^{\infty }\left(e^{-G\left(y,z|v^{n}\right)}\right)}\frac{\partial }{\partial \theta }G\left(y,z|v^{n}\right)\\ & =\sum_{z=1}^{\infty }P\left(z|v^{n},y\right)\frac{\partial }{\partial \theta }G\left(y,z|v^{n}\right) \end{array}$$

In order to compute (A1), we need to compute $\frac{\partial }{\partial \theta }G\left(y,z|v^{n}\right)$ first, which are shown as follows:

(A2) $$\begin{array}{cc} \frac{\partial }{\partial W_{i\cdot }}G(y,z|v) & =-\frac{\partial }{\partial W_{i\cdot }}\sum_{i^{\prime }=1}^{z}\ln\left(1+\exp\left(W_{i^{\prime }\cdot }v+U_{i\cdot }e_{y}+c_{i^{\prime }}\right)\right)-\beta_{i^{\prime }}\\ & =-\frac{\partial }{\partial W_{i\cdot }}\sum_{i^{\prime }=1}^{z}\ln\left(1+\exp\left(W_{i^{\prime }\cdot }v+U_{i\cdot }e_{y}+c_{i^{\prime }}\right)\right)-\beta_{i^{\prime }}\\ & =-H\left(z-i\right)\frac{\partial }{\partial W_{i\cdot }}\ln\left(1+\exp\left(W_{i\cdot }v+U_{i\cdot }e_{y}+c_{i}\right)\right)\\ & =-H\left(z-i\right)\frac{\exp\left(W_{i\cdot }v+U_{i\cdot }e_{y}+c_{i}\right)}{1+\exp\left(W_{i\cdot }v+U_{i\cdot }e_{y}+c_{i}\right)}v\\ & =-H\left(z-i\right)s\left(W_{i\cdot }v+U_{i\cdot }e_{y}+c_{i}\right)v \end{array}$$

Similarly:

(A3) $$\frac{\partial G\left(y,z|v\right)}{\partial U_{i\cdot }}=-H\left(z-i\right)s\left(W_{i\cdot }v+U_{i\cdot }e_{y}+c_{i}\right)e_{y}$$

(A4) $$\frac{\partial }{\partial d}G\left(y,z|v\right)=-H\left(z-i\right)e_{y}$$

(A5) $$\frac{\partial }{\partial c_{i}}G\left(y,z|v\right)=-H\left(z-i\right)s\left(W_{i\cdot }v+U_{i\cdot }e_{y}+c_{i}\right)$$

where:

$$H\left(z-i\right)=\left\{ \begin{array}{ll} 0, & z<i\\ 1, & z\geq i \end{array}\right.$$

substituting (A2)–(A5) into (A1), we get:

(A6) $$\begin{array}{ll} \frac{\partial F\left(y|v\right)}{\partial W_{i\cdot }} & =-\sum_{z=1}^{\infty }P\left(z|v,y\right)H\left(z-i\right)s\left(W_{i\cdot }v+U_{i\cdot }e_{y}+c_{i}\right)v\\ & =P\left(z\geq i|v,y\right)s\left(W_{i\cdot }v+U_{i\cdot }e_{y}+c_{i}\right)v \end{array}$$

(A7) $$\begin{array}{ll} \frac{\partial F\left(y|v\right)}{\partial U_{i\cdot }} & =-\sum_{z=1}^{\infty }P\left(z|v,y\right)H\left(z-i\right)s\left(W_{i\cdot }v+U_{i\cdot }e_{y}+c_{i}\right)e_{y}\\ & =P\left(z\geq i|v,y\right)s\left(W_{i\cdot }v+U_{i\cdot }e_{y}+c_{i}\right)e_{y} \end{array}$$

(A8) $$\frac{\partial F\left(y|v\right)}{\partial d}=-\sum_{z=1}^{\infty }P\left(z|v,y\right)e_{y}=-e_{y}$$

(A9) $$\begin{array}{ll} \frac{\partial F\left(y|v\right)}{\partial c_{i}} & =-\sum_{z=1}^{\infty }P\left(z|v,y\right)H\left(z-i\right)s\left(W_{i\cdot }v+U_{i\cdot }e_{y}+c_{i}\right)\\ & =P\left(z\geq i|v,y\right)s\left(W_{i\cdot }v+U_{i\cdot }e_{y}+c_{i}\right) \end{array}$$

where, $P\left(z\geq i|v,y\right)=1-\sum_{z^{\prime }=1}^{i}P\left(z^{\prime }|v,y\right)$.
